# Long-Term Outcomes after Rotational Atherectomy for Calcified Chronic Total Occlusion versus Nonchronic Total Occlusion Coronary Lesions

**DOI:** 10.1155/2022/2593189

**Published:** 2022-12-27

**Authors:** Karim Elbasha, Nader Mankerious, Mohamed Alawady, Ghada Ibrahim, Radwa Abdullah, Mohamed Abdel-Wahab, Rayyan Hemetsberger, Ralph Toelg, Gert Richardt, Abdelhakim Allali

**Affiliations:** ^1^Cardiology Department, Heart Center Segeberger Kliniken GmbH, Bad Segeberg, Germany; ^2^Cardiology Department, Zagazig University, Sharkia, Egypt; ^3^Cardiology Department, Heart Center Leipzig at the University of Leipzig, Leipzig, Germany; ^4^Department of Cardiology, Internal Medicine II, Medical University of Vienna, Austria; ^5^Medical Clinic II, University Heart Center Lubeck, Lubeck, Germany

## Abstract

**Background:**

The role of rotational atherectomy (RA) in contemporary percutaneous coronary intervention (PCI) is expanding to include certain chronic total occlusion (CTO) lesions. However, the long-term outcome of RA in CTOs is still unclear.

**Objective:**

To investigate in-hospital and long-term outcomes after RA for CTO compared to non-CTO calcified lesions. Moreover, this report evaluates the role of the elective RA approach in calcified CTOs.

**Methods and Results:**

This study enrolled 812 patients (869 lesions; CTO, *n* = 80 versus non-CTO, *n* = 789). The mean age of the study population was 73.1 ± 8.6 years, the baseline characteristics were comparable in both groups. Balloon-resistant CTO lesions represented the main indication for RA in CTO patients (61.2%). The mean J-CTO score was 2.42 ± 0.95. The angiographic success rate was lower in CTO patients (88.8% vs 94.9%; *p* = 0.022). In-hospital major adverse cardiac events (MACE) rate was comparable in both groups (CTO 8.8% vs 7.0% in non-CTO;*p* = 0.557). At two-year follow-up, a higher target lesion failure (TLF) was observed in CTO group (25.5% vs 15.1%, log rank *p* = 0.041), driven by higher cardiac mortality while the clinically driven target lesion revascularisation (TLR) was comparable between the study groups. Elective RA for CTO had a shorter procedural time and lower rate of dissection (7.5% vs 25%; *p* = 0.030) compared to bail-out RA with similar long-term outcomes.

**Conclusion:**

Compared to non-CTO, RA for CTO can be performed with a high procedural success rate and comparable in-hospital outcomes. Apart from higher cardiac mortality in the CTO group, the long-term outcomes are comparable in both groups. Elective RA is a feasible and beneficial approach to be used in CTO intervention.

## 1. Introduction

Chronic total occlusion (CTO) is encountered in approximately 15–20% of all percutaneous coronary interventions (PCI) [1]. Calcified CTO lesions are not uncommon, since moderate or severe calcification is present in more than half of CTO lesions [2].

Indeed, wire crossing through the CTO lesion is not the only challenging step during CTO PCI as balloon-resistant calcified CTO is another difficulty and it includes balloon uncrossable and undilatable lesions. Balloon uncrossable lesions are those lesions that cannot be crossed with the smallest available balloons after successful guidewire crossing, and it ranges from 6% to 9% of all CTOs, while balloon undilatable lesions are those that cannot be expanded despite using high-pressure balloons and it represents about 12% [3].

Rotational atherectomy (RA) became an indispensable tool for treating calcified coronary lesions and it is increasingly applied for the preparation of certain calcified CTOs. Elective application of RA in calcified CTOs is not a common approach and using RA in CTO is usually limited to balloon-resistant calcified lesions. However, RA in calcified CTOs could be beneficial when used electively as being preprocedurally or early intraprocedurally planned [4].

Altogether, heavily calcified balloon-resistant CTO lesions are among the most challenging coronary lesions as they often require sophisticated techniques and unfortunately are associated with lower success rates and more complications [2]. The feasibility and in-hospital outcomes of RA in CTOs were reported in some studies [5, 6]. However, data regarding the long-term outcomes of a calcified CTO lesion treated with RA, particularly when compared with non-CTO, are lacking. Furthermore, the role of elective RA in CTO lesions is unclear.

## 2. Methods

### 2.1. Study Design and Patients

This is a retrospective analysis of patients who underwent RA at a single centre (Heart Centre, Segeberger Kliniken, Bad Segeberg, Germany) between 2004 and 2018. We analysed 869 RA procedures for 812 patients; 789 non-CTO lesions (*n* = 732 patients); and 80 CTO lesions (*n* = 80 patients). Baseline, procedural, in-hospital, and long-term follow-up data were compared. Written informed consent was obtained from all patients for analysis of their anonymized data, and data collection were approved by the local ethics committee.

### 2.2. Rotational Atherectomy

RA was applied in heavily calcified lesions according to the operator's discretion as either an elective or a bail-out strategy (including balloon-resistant CTO lesions). In CTO lesions, wire exchange (to Rota-Wire) was performed by using a microcatheter, which either crossed the lesion or was advanced as far as possible into the lesion (in cases when a microcatheter could not completely cross the lesion). A procedure was regarded as a RA as soon as the Rota-Wire was advanced through the target lesion. RA was performed using the Rotablator or Rotapro systems (Boston Scientific Scimed, Inc., Maple Grove, MN, USA). The burr size was chosen up to 0.7 of the vessel diameter for non-CTO RA but a smaller burr size was used for CTO lesions [7]. Prior to RA, patients were treated with 325–500 mg aspirin orally and an oral loading dose of a P2Y12 inhibitor (clopidogrel in patients presenting with chronic coronary syndrome/prasugrel, or ticagrelor in patients presenting with acute coronary syndrome) and periprocedural anticoagulation with either UFH or bivalirudin was routinely administered. Post-PCI dual antiplatelet therapy and duration were applied according to the presentation (chronic coronary syndrome or acute coronary syndrome) as recommended in the guidelines.

### 2.3. Definitions

A CTO was defined as a coronary occlusion with thrombolysis in myocardial infarction (TIMI) 0 flow for ≥3 months. The duration of CTO was either certain (angiographically confirmed) or likely (clinically confirmed) [8]. For CTO lesions, the J-CTO score was calculated as one point was assigned to each of the following variables: previously failed attempt, blunt stump, calcification, bending >45°, and occlusion length ≥20 mm [9]. Calcification was assessed angiographically according to the Mintz classification [10].

Angiographic success was defined as RA followed by stent implantation with residual stenosis <30% and TIMI 3 flow in the target vessel at the end of the procedure. The procedural success was defined as angiographic success with the absence of in-hospital periprocedural complications (all-cause death, periprocedural MI, recurrent angina requiring target vessel revascularisation (TVR) with PCI or emergency coronary artery bypass graft (CABG), and tamponade requiring pericardiocentesis or surgery) [11]. Bail-out RA was defined as a procedure, where the decision to perform RA was made during the PCI after the failure of balloon-based angioplasty. Elective RA was either preprocedurally or early intraprocedurally planned, based on angiographic or intravascular imaging but before any attempt of balloon angioplasty. Early generation drug-eluting stents (DESs) represent an early experience of DESs with thick struts platform and polymer that elutes antiproliferative medication, including the sirolimus-eluting CypherTM stent (Cordis, Miami Lakes, FL, USA) and the paclitaxel-eluting Taxus Liberte TM stent (Boston Scientific, Boston, MA, USA). While new-generation drug-eluting stents are characterized by thinner stent strut platforms, polymers with improved biocompatibility and limus-based antiproliferative agents, including the cobalt-chromium everolimus-eluting XienceTM stent (Abbott Vascular, Santa Clara, CA, USA), the platinum-chromium everolimus-eluting PromusTM stent (Boston Scientific, Natick, MA, USA), as well as the sirolimus-eluting OrsiroTM stent (Biotronik, Bülach, Switzerland). The periprocedural myocardial infarction (MI) (type 4a MI) was defined according to the fourth universal definition of MI [12]. Coronary perforation was defined and classified according to Ellis classification [13]. Slow flow is defined as TIMI flow grade ≤2 at the end of the procedure [14].

### 2.4. Follow-Up

Clinical follow-up was obtained either by on-site clinical visits or scripted telephone calls with the patients or their general practitioners (our centre applies a routine angiographic and clinical follow-up after complex interventions). In-hospital MACE was defined as the composite of all-cause mortality, periprocedural MI and TVR, and two-year target lesion failure (TLF) was defined as the composite of cardiac death, target vessel MI, and clinically driven target lesion revascularisation (TLR). The target lesion was defined as the treated segment including the 5 mm margin proximal and distal to the stent [15].

### 2.5. Statistical Analysis

Qualitative variables are summarized as frequencies and percentages, while quantitative variables are summarized as mean ± SD or median (25^th^–75^th^ quartiles), depending on variables distribution. For continuous data, Student's *t*-test or nonparametric tests (Mann–Whitney U) were used for the comparison according to data distribution, while chi-square or Fischer's exact tests were used for the comparison of qualitative data. Survival curves were created using the Kaplan–Meier method and compared using log rank test. Univariate analysis was performed using Cox regression analysis, the hazard ratio (HR) and the 95% confidence interval (CI) were presented. Multivariate Cox regression analyses were performed using entry criteria of *p* < 0.1 in the univariate analysis. Statistical analysis was performed using SPSS v. 23 software (IBM, Armonk, NY, USA).

## 3. Results

### 3.1. Baseline Patient Characteristics

Clinical characteristics of both groups are summarized in Table 1. The mean age of the whole study population was 73.1 ± 8.6 years, 30.1% of patients had a reduced left ventricular ejection fraction (LV-EF<50%), with a high proportion of previous CABG (19.6%), diabetes mellitus (DM) (35.3%), and three vessel disease (59.6%). CTO patients had a trend towards lower LV EF (49.6 ± 12.8 vs 52.1 ± 12.6; *p* = 0.098) and higher rate of previous PCI (48.8% vs 38.7%; *p* = 0.080).

### 3.2. Angiographic Characteristics and Procedural Details

The main angiographic and procedural characteristics of both groups are listed in Table 2. Bail-out RA was the main strategy of CTO RA (61.3% vs 38.5%; *p* <  0.001), where 35% of the lesions were balloon uncrossable and 26.3% were balloon undilatable. The mean J-CTO score was 2.42 ± 0.95 and 36.4% of patients had a J-CTO score ≥3. Antegrade wire escalation was the main approach of CTO PCI (95%). CTO lesions were treated with longer stents (64 mm (IQR 41–94) vs 32 mm (IQR 20–50); *p* < 0.001) and smaller burr size (1.38 ± 0.19 vs 1.53 ± 0.21 mm; *p* < 0.001). Additionally, CTO PCI took longer time (124 min (IQR 97–170) vs 79 min (IQR 58–113); *p* < 0.001) and consumed higher amount of contrast (304 ml (IQR 244–378) vs 200 ml (IQR 150–300); *p* < 0.001).

### 3.3. Periprocedural Complications and In-Hospital Outcome

In-hospital outcomes and complications are outlined in Tables 3 and 4. The angiographic success was achieved in 71 CTO RA procedures (88.8% vs 94.9%; *p* = 0.022), and procedural success rate was 80% in CTO versus 90.5% in non-CTO RA procedures (*p* = 0.003).

In-hospital MACE was comparable in both groups (8.8% vs 7%; *p* = 0.557). However, the incidence of slow flow (7.5% vs 2.9%; *p* = 0.030), coronary perforation (8.8% vs 2%; *p* < 0.001), and cardiac tamponade (5.0% vs 1.8%; *p* = 0.054) were higher in the CTO group. Coronary perforation (66.7%) was the main cause of cardiac tamponade, followed by the insertion of temporary pacemaker (27.8%). Coronary perforation occurred mainly because of high pressure inflation of the balloons/stents (43.5%) followed by PCI wire perforation in 30.4%. Seven wire perforations were detected; 2 in CTO group (one due to Pilot 150 guidewire and the other due to the Rotawire), while in non-CTO group 5 wire perforation were detected (2 from the Rotawire, 2 by Whisper, and one by Pilot 50 guidewires). All wire-induced perforations were caused by unintentional pushing of the wires to very distal and/or in small branches.

### 3.4. Long-Term Outcome

**Figure 1:**
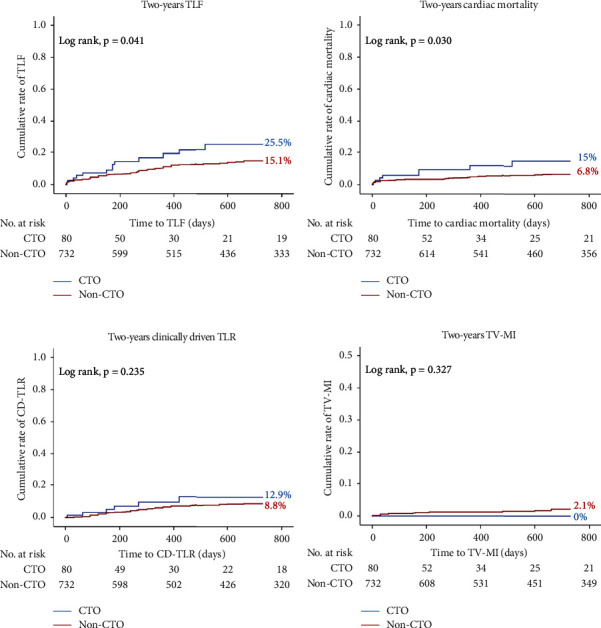
Kaplan–Meier curves of the two-year cumulative rate of the occurrence of the endpoint. (a) The cumulative rate of the 2-year target lesion failure (TLF), (b) cardiac mortality, (c) clinically driven target lesion revascularisation (TLR), and (d) target vessel myocardial infarction (TV MI) between chronic total occlusion (CTO) and nonchronic total occlusion (non-CTO) groups.

At two-year follow-up, we observed a higher TLF in the CTO group (25.5% vs 15.1%; log rank *p* = 0.041), that was mainly driven by higher cardiac deaths (15.0% vs 6.8%; log rank *p* = 0.030). The 2-year target vessel MI and clinically driven TLR were comparable between the two groups (target vessel MI: 0% vs 2.1%; log rank *p* = 0.327 and clinically driven TLR: 12.9% vs 8.8%; log rank *p* = 0.235) (Figure 1). The 2-year clinically driven TVR was also comparable in both the study groups (CTO: 16.7% vs non-CTO: 10.5%; log rank *p* = 0.428). On multivariate regression analyses, the presence of CTO lesion, chronic kidney disease (CKD), periprocedural MI, and reduced LV-EF were independently associated with the two-year TLF.  Table 5 lists the uni- and multivariate regression analyses of 2-year TLF predictors.

### 3.5. Elective versus Bail-Out RA in the CTO Group

**Figure 2:**
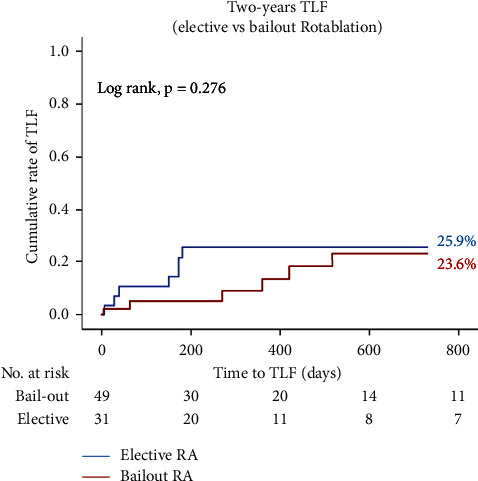
Kaplan–Meier curves of the two-year target lesion failure (TLF) rate. The cumulative rate of the 2-year target lesion failure (TLF) in bail-out versus elective rotational atherectomy (RA) for chronic total occlusion (CTO) lesions.

In the CTO group, 31 patients (38.7%) were treated with elective RA while in the remaining 49 patients (61.3%), RA was performed as bail-out (due to either balloon uncrossable or undilatable lesions). Compared to the bail-out RA, elective procedures took shorter time (150 min (IQR 114–180) vs 115 min (IQR 80–137); *p* = 0.003), had lower rate of dissections (25% vs 7.5%; *p* = 0.030), and less frequently required two or more burrs (11.2% vs 1.3%; *p* = 0.043) as outlined in Table 6. However, the estimated rate of 2-year TLF was not significantly different (25.9% vs 23.6%; log rank, *p* = 0.276) (Figure 2).

## 4. Discussion

The main findings of our study are as follows: (1) RA for CTO lesions was achieved with a high success rate. However, the overall complications were higher than non-CTO RA, (2) long-term follow-up revealed higher TLF in the CTO patients after RA, and (3) elective RA could be performed in calcified CTO lesions with shorter procedure time and less dissections than bail-out procedures but with similar long-term outcome.

Rotational atherectomy has a unique mechanism for treating calcified lesions that differs from other tools such as orbital atherectomy, lithotripsy, and cutting balloons. It utilizes a rotating diamond tipped burr with a constant, circular orbit that ablates in the forward direction, in other words it creates its own pathway [16]. Therefore, RA appears to be the most suitable plaque modifying tool for treating balloon resistant CTO lesions, particularly balloon uncrossable lesions that prevent passage of the smallest available balloon, since the other available tools require lesion crossing first.

Most available data on RA in CTO compared the feasibility and in-hospital outcomes in rotablated CTOs versus conventional (non-rotablated) CTO PCI. However, such comparison might be inappropriate and biased, as patients who need RA are usually older, have more comorbidities and more calcified long lesions (higher J-CTO score) [17]. Moreover, all rotablated CTOs had a successful wire crossing (according to studies definition), so it is not appropriate to compare the success rate of rotablated CTOs with other CTO PCIs that include lesions with a failure of wiring. Our study compared the outcome in CTO and non-CTO calcified lesions that required RA, and so the baseline characteristics were nearly similar in both groups.

The procedural success rate after RA for CTOs varies in the literature from 77% to 95.6% [6, 18, 19]. In our analysis, CTO RA showed lower success rate than non-CTO and it was mainly driven by the presence of more slow flow at the end of the procedure. However, it is comparable to the success rate in the previously mentioned studies. The high rate of slow flow in our cohort could be attributed to the aggressive RA (using large burr und higher speed), so we recommend to use smaller burr (preferably 1.25 mm) and lower speed (140000 instead of 170000 RPM) in CTO RA to avoid slow flow. Additionally, the use of rotational atherectomy cocktail (nitro-glycerine, verapamil, and heparin) with performing short runs (20–30 s) might reduce the rate of slow flow. It is noteworthy that early generation DESs were more used in non-CTO group, while the newer generation DESs were more implanted in CTO patients, this discrepancy attributed to the low number of CTO patients who required RA in this time period (era of early generation DESs) in our centre (only 1.4% of patients with CTO Vs 21% with non-CTO were treated by RA before 2007). Additionally, the advancement of CTO tools and increasing the experience in dealing with such complex CTO lesions synchronised with the development of newer generation DES.

The complication rate was clearly higher in the CTO group in the form of coronary perforation, slow flow, and tamponade. Interestingly, the main causes of these complications were not directly related to RA; coronary perforation occurred mainly after a high-pressure inflation of balloons/stents, and due to PCI wire perforation, while direct burr-induced perforation only occurred in 4 cases (2 cases in each group). The perforation rate in our CTO group was higher than that mentioned in other previous studies by Azzalini et al. [18] and Brinkmann et al. [19] that could be related to heavily calcified lesions in older patients in our cohort and the aggressive lesion preparation so that it is preferred to avoid very high pressure inflation of the balloons/stents after rotablation in order to avoid perforation. Additionally, temporary pacemaker insertion before RA was the second common cause of cardiac tamponade after coronary perforation.

Elective RA is not a common approach for preparation of CTO lesions and the most common indication to rotablate the CTO is the presence of balloon-resistant lesions. However, in our study, 38.7% of CTO RA were performed electively due to the presence of angiographic severe calcification [10]. The main benefits of elective RA versus bail-out were shorter procedure time and lower rate of coronary dissection. However, these benefits were not reflected on the long-term outcome as both approaches had similar two-year TLF rate. These results are in the same line with the study by Allali et al. [20] that compared planned versus bail-out RA for heavily calcified coronary lesions.

Data about long-term outcomes after RA in CTOs are scarce, only two studies compared the long-term outcome in rotablated CTO versus conventional CTO PCI, both included a relatively small number of CTO patients [18, 21]. To the best of our knowledge, our study is the first to report the long-term outcome after RA in CTO compared to non-CTO and to evaluate the role of elective RA in calcified CTO lesions.

Our study reported a higher 2-year TLF rate in CTO patients that was driven by higher cardiac mortality. Although both the study groups had similar baseline characteristics, the CTO patients had a trend toward lower LV-EF. Moreover, higher cardiac mortality could be related to the higher rate of periprocedural complications in the CTO group, higher perforation, and tamponade rate in addition to a trend toward higher periprocedural MI. The higher rate of perforation and tamponade might be associated with poor long-term survival as reported by Stathopoulos et al. [22]. Furthermore, slow flow at the end of CTO PCI procedure is associated with higher MACE according to the report by Wang et al. [14] and as previously mentioned, we can limit these complications by less aggressive balloon/stent dilation after RA and proper selection of burr size and speed. A multicentre study to identify a survival advantage score to predict the outcome after CTO PCI, reported that the association between three variables (multivessel disease, Canadian cardiovascular society (CCS) score ≥2, and previous MI) in CTO patients predict poor long-term outcome after CTO revascularisation [23]. In our CTO cohort, multivessel disease was present in 68.8% and about 58.7% had CCS ≥ 2, and 21.3% reported previous MI. This could be another explanation for higher cardiac mortality in CTO patients even after revascularisation.

It is worth mentioning that 2-year clinically driven TLR, clinically driven TVR, and target vessel MI were comparable in both groups which are considered important long-term effectiveness endpoints of RA for calcified CTO lesions.

The major limitation of this study is its nonrandomized design in which operator bias and unmeasured confounders may prohibit definitive conclusions. This study is also retrospective and has been performed at a single centre. Moreover, the relatively small size of CTO RA group might have affected the statistical comparison of in-hospital complications and the long-term outcome during follow-up. However, our report still provides relevant data on the real-world utilization and outcomes of RA in a cohort of CTO patients.

## 5. Conclusion

Compared to non-CTO, RA in CTO is feasible with a high success rate and similar in-hospital outcomes but with higher periprocedural complications. Apart from higher cardiac deaths in CTO patients after RA due to higher clinical and procedural complexities, the long-term outcomes were comparable. Elective RA in CTO can shorten the procedure time and decrease the incidence of dissection in comparison with the bail-out RA.

## Figures and Tables

**Table 1:** Baseline clinical characteristics of the study population.

	Total (*n* = 812)	CTO RA (*n* = 80)	Non-CTO RA (*n* = 732)	*p* value
Age (years)	73.1 ± 8.6	72.5 ± 8.9	73.1 ± 8.57	0.567
Gender				
Male (%)	650 (80%)	64 (80%)	586 (80.1%)	0.991
DM (%)	287 (35.3%)	32 (40%)	255 (35.1%)	0.383
HTN (%)	727 (89.5%)	74 (92.5%)	653 (90.2%)	0.506
Dyslipidemia (%)	514 (63.3%)	55 (68.8%)	459 (63.4%)	0.344
Smoking (%)	244 (30%)	24 (30%)	220 (30.4%)	0.943
Previous CABG (%)	159 (19.6%)	16 (20%)	143 (19.5%)	0.919
Previous MI (%)	169 (20.8%)	17 (21.3%)	152 (20.8%)	0.921
Previous PCI (%)	322 (39.7%)	39 (48.8%)	283 (38.7%)	0.080
Number of CAD (%)				
One vessel	106 (13.1%)	8 (10%)	98 (13.4%)	0.214
Two vessels	222 (27.3%)	17 (21.3%)	205 (28%)	
Three vessels	484 (59.6%)	55 (68.8%)	429 (58.6%)	
CKD (%)	129 (15.9%)	7 (8.8%)	122 (16.7%)	0.066
Dialysis (%)	11 (1.4%)	2 (2.5%)	9 (1.2%)	0.351
Left ventricular EF	51.8 ± 12.6	49.6 ± 12.8	52.1 ± 12.6	0.098
Left ventricular	243 (30.1%)	27 (33.8%)	216 (29.7%)	0.455
EF < 50% (%)				

Data presented as mean ± standard deviation or number and percentage. CABG, coronary artery bypass graft operation; CAD, coronary artery disease; CKD, chronic kidney disease; CTO, chronic total occlusion; DM, diabetes mellitus; EF, ejection fraction; HTN, hypertension; MI, myocardial infarction; PCI, percutaneous coronary intervention; RA, rotational atherectomy.

**Table 2:** Angiographic and procedural characteristics.

	Total (*n* = 869)	CTO RA (*n* = 80)	Non-CTO RA (*n* = 789)	*p* value
Indication for PCI (%)				
Stable CAD	652 (75%)	59 (73.8%)	593 (75.2%)	0.432
UA	109 (12.5%)	14 (17.5%)	95 (12%)	
NSTEMI	82 (9.4%)	5 (6.3%)	77 (9.8%)	
STEMI	26 (3%)	2 (2.5%)	24 (3%)	
Target vessel (%)				
LAD	384 (44.2%)	23 (28.7%)	361 (45.8%)	<0.001
LCX	124 (14.3%)	7 (8.8%)	117 (14.8%)	
LM	95 (10.9%)	0	95 (12%)	
RCA	266 (30.6%)	50 (62.5%)	216 (27.4%)	
Ostial lesion (%)	223 (25.7%)	11 (13.8%)	212 (26.9%)	0.012
Bifurcation lesion (%)	347 (39.9%)	20 (25%)	327 (41.4%)	0.004
Indication for RA (%)				
Elective	516 (59.4%)	31 (38.7%)	485 (61.5%)	<0.001
Bail-out	353 (40.6%)	49 (61.3%)	304 (38.5%)	
Burr size (mm)	1.51 ± 0.21	1.38 ± 0.19	1.53 ± 0.21	<0.001
Number of burrs (%)				
One burr	721 (84.5%)	69 (86.3%)	652 (84.2%)	0.473
Two burrs	132 (15.5%)	10 (12.5%)	122 (15.8%)	
Number of stents	1.96 ± 1.03	2.64 ± 1.37	1.83 ± 0.97	<0.001
Stent type (%)				<0.001
Early generation	288 (33.1%)	13 (16.3%)	275 (34.9%)	
New generation	433 (49.8%)	59 (73.7%)	374 (47.4%)	
Total stent length (mm)	34 [22–53]	64 [41–94]	32 [20–50]	<0.001
Maximum stent size (mm)	3.05 ± 0.53	2.92 ± 0.94	3.06 ± 0.46	0.210
Postdilatation (%)	558 (64.2%)	46 (57.5%)	512 (64.9%)	0.194
Procedure time (min)	83 [60–119]	124 [97–170]	79 [58–113]	<0.001
Radiation time(min)	25 [16–39]	54.5 [38–86]	23.5 [15–35]	<0.001
Contrast dose (ml)	200 [150–300]	304 [244–378]	200 [150–300]	<0.001
Hemodynamic support (%)				0.931
Impella	14 (1.6%)	1 (1.3%)	13 (1.6%)	
IABP	18 (2.1%)	2 (2.5%)	16 (2%)	

Data presented as mean ± standard deviation or number and percentage. CAD, coronary artery disease; CTO, chronic total occlusion; IABP, intra-aortic balloon counterpulsation; LAD, left anterior descending; LCX, left circumflex; LM, left main coronary arteries; NSTEMI, non-ST-segment elevation myocardial infarction; RA, rotational atherectomy; RCA, right coronary artery; STEM, ST-segment elevation myocardial infarction; UA, unstable angina.

**Table 3:** In-hospital outcomes.

	Total (*n* = 812)	CTO RA (*n* = 80)	Non-CTO RA (*n* = 732)	*p* value
In-hospital MACE (%)	58 (7.1%)	7 (8.8%)	51 (7%)	0.557
In-hospital death (%)	18 (2.2%)	3 (3.8%)	15 (2%)	0.327
Cardiac	18 (2.2%)	3 (3.8%)	15 (2%)	
Noncardiac	0 (0%)			
TVR	7 (0.7%)	1 (1.3%)	6 (0.8%)	0.693
Periprocedural MI (%)	42 (5.2%)	7 (8.8%)	35 (4.8%)	0.128

Data presented as number and percentage. CTO, chronic total occlusion; MACE, major adverse cardiac events; MI, myocardial infarction; RA, rotational atherectomy; TPM, temporary pacemaker; TVR, target vessel revascularisation.

**Table 4:** Success rate and periprocedural complications.

	Total (*n* = 869)	CTO RA (*n* = 80)	Non-CTO RA (*n* = 789)	*p* value
Angiographic success (%)	820 (94.4%)	71 (88.8%)	749 (94.9%)	0.022
Procedural success (%)	778 (89.5%)	64 (80%)	714 (90.5%)	0.003
Slow flow (%)	29 (3.3%)	6 (7.5%)	23 (2.9%)	0.030
Coronary perforation (%)	23 (2.6%)	7 (8.8%)	16 (2%)	<0.001
Covered stent	5 (0.6%)	3 (3.8%)	2 (0.3%)	0.007
Tamponade (%)	18 (2.1%)	4 (5%)	14 (1.8%)	0.054
Pericardiocentesis	17 (2%)	4 (5%)	13 (1.6%)	0.039

Data presented as number and percentage. CTO, chronic total occlusion; RA, rotational atherectomy.

**Table 5:** Univariate and multivariate regression analyses identifying predictors of the long-term target lesion failure (TLF).

	Univariate analysis; hazard ratio (95% CI)		*p* value	Multivariate analysis; hazard ratio (95% CI)		*p* value
Age	1.02	(0.99–1.04)	0.180			
Male sex	1.23	(0.79–1.91)	0.367			
DM	1.763	(1.19–2.59)	0.004	1.47	(0.99–2.18)	0.056
HTN	0.564	(0.33–0.98)	0.041	0.53	(0.31–0.93)	0.026
Smoking	1.275	(0.86–1.91)	0.235			
Dyslipidemia	1.09	(0.72–1.65)	0.696			
CKD	1.69	(1.07–2.69)	0.025	1.72	(1.08–2.74)	0.023
EF < 50%	1.78	(1.20–2.63)	0.004	1.59	(1.07–2.34)	0.023
CTO lesion	1.82	(1.02–3.26)	0.044	2.04	(1.13–3.69)	0.018
Multivessel disease	1.74	(0.88–3.45)	0.110			
Periprocedural MI	2.72	(1.46–5.09)	0.002	2.46	(1.28–4.76)	0.007
Slow flow	1.82	(0.79–4.14)	0.156			
Perforation	1.69	(0.62–4.58)	0.306			
Bifurcation	1.12	(0.76–1.654)	0.562			
Ostial lesion	1.41	(0.94–2.13)	0.098	1.25	(0.82–1.92)	0.297
Stent length	1.00	(0.99–1.01)	0.525			
Bailout RA	1.08	(0.73–1.59)	0.712			
Early generation DES	0.957	(0.62–1.48)	0.845			

Data are presented as hazard ratio and 95% confidence interval. CAD, coronary artery disease; CKD, chronic kidney disease; CTO, chronic total occlusion; DES, drug-eluting stent; DM, diabetes mellitus; EF, ejection fraction; GFR, glomerular filtration rate; HTN, hypertension; MI, myocardial infarction; PCI, percutaneous coronary intervention; RA, rotational atherectomy.

**Table 6:** Periprocedural complications and in-hospital outcomes after bail-out versus elective RA within the CTO group.

	Total (*n* = 80)	Bailout RA (*n* = 49)	Elective RA (*n* = 31)	*p* value
Angiographic success	71 (88.8%)	42 (85.7%)	29 (93.5%)	0.280
In-hospital MACE (%)	7 (8.8%)	4 (5.0%)	3 (3.8%)	0.557
Periprocedural MI (%)	7 (8.8%)	4 (5%)	3 (3.8%)	0.557
Perforation (%)	7 (8.8%)	4 (5%)	3 (3.8%)	0.815
Tamponade (%)	4 (5.0%)	1 (1.3%)	3 (3.7%)	0.127
Slow flow (%)	6 (7.5%)	4 (5.0%)	2 (2.5%)	0.777
Presence of dissection	26 (32.5%)	20 (25%)	6 (7.5%)	0.030
Contrast dose (ml)	304 [244–378]	300 [250–411]	300 [210–376]	0.292
Procedure time (min)	124 [97–170]	150 [114–180]	115 [80–137]	0.003
Radiation time (min)	54.5 [38–86]	57 [37–80]	49 [33–116]	0.741
Number of burrs				
1	69 (86.3%)	39 (48.8%)	30 (37.5%)	0.043
2	10 (12.5%)	9 (11.2%)	1 (1.3%)	

Data presented as mean ± standard deviation or number and percentage. MACE, major adverse cardiac events; MI, myocardial infarction; RA, rotational atherectomy.

## Data Availability

The data used to support the findings of this study will be available from the corresponding author upon request.

## Additional Points

*Perspectives.* What is known? RA for preparation of calcified CTO lesions is feasible with high procedural success rate. What is new? Up to our knowledge, this is the first study to compare the long-term outcome after RA in CTO versus non-CTO calcified lesions and to evaluate the role of elective RA in calcified-resistant CTO lesions. This study emphasis on the importance of RA in certain CTO lesions and gives insights into the long-term outcomes compared to the conventional RA for calcified non-CTOs. Moreover, it sheds the light on elective RA for CTOs and its advantages over bail-out RA.
